# Cro-Magnons Conquered Europe, but Left Neanderthals Alone

**DOI:** 10.1371/journal.pbio.0020449

**Published:** 2004-11-30

After miners unearthed a skull and bones in a Neander Valley cave in Germany in 1856—three years before the publication of *On the Origin of Species*—the remains were initially described as either those of a “brutish” race or of someone disfigured by disease. As Darwinian evolution caught on, so did the realization that these fossils were evidence of an earlier human species. Scientists have been debating Neanderthal's place in human evolution ever since.

An ongoing question concerns the possibility that Neanderthals and early humans mated, since they likely crossed paths during thousands of years of European cohabitation. In a new study, Mathias Currat and Laurent Excoffier present a simulation model based on what we know about the population density and distribution of Neanderthals and Cro-Magnons. Their results complement recent genetic and morphological evidence indicating that early human and Neanderthal interbreeding was unlikely.

The notion that modern Europeans directly descended from Neanderthals has mostly yielded to two competing models: One postulates that modern humans arose in Africa about 130,000 years ago and completely replaced coexisting archaic forms with no interbreeding, while the other proposes a gradual transition with interbreeding.

Though mounting genetic evidence (based on mitochondrial DNA extracted from fossils) suggests Neanderthals and early humans did not breed, the evidence has been inconclusive. It's possible, for example, that any Neanderthal gene “leakage” could have been lost through genetic drift if the mating populations were small. And because so few fossils are available to analyze, previous studies could rule out only Neanderthal contributions over 25%.

Currat and Excoffier's model refines various parameters—such as geographic boundaries, local population variations, range expansion, and competition for resources—based on archeological and demographic data for both populations. Evidence suggests modern humans replaced Neanderthals over 12,500 years, for example, which constrains the speed at which modern humans could expand.

The authors started with a scenario based on a set of “plausible” parameter values—their basic scenario—and then varied the local interbreeding rate and, for example, the population size and location of Cro-Magnons, to produce eight alternate scenarios describing how Cro-Magnon colonization of Europe might have proceeded. They estimated the likely proportion of Neanderthal gene contributions to the modern gene pool using “coalescent simulations,” which generate the genealogies and diversity of genes in local populations based on simulations of their population densities and migration histories. The simulations show that if Neanderthals bred with Cro-Magnons without constraints over thousands of years, Neanderthal contributions to the modern gene pool “would be immense.” Surprisingly, the simulations also show that even a very small mixing should lead to high levels of Neanderthal DNA in modern humans.[Fig pbio-0020449-g001]

**Figure pbio-0020449-g001:**
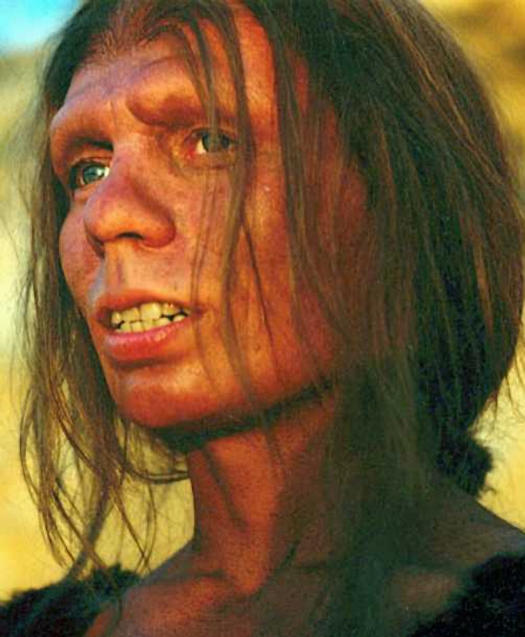
Reconstruction of Neanderthal woman

What could account for this counterintuitive result? Given a low population density with small local breeding populations, any introduction of Neanderthal genes would decrease the frequency of Cro-Magnon genes of that population; if these Neanderthal integrations take place as the Cro-Magnon population is expanding, newly acquired Neanderthal genes would also be amplified

Since no Neanderthal mitochondrial DNA has been found in modern-day Europeans, the authors modeled the maximum number of interbreeding events that would support this observation. The estimated maximum number of events, it turns out, falls between 34 and 120—extremely low values, Currat and Excoffier conclude, “given the fact that the two populations must have coexisted for more than 12,000 years.”

While the authors acknowledge their simulations suggest rather than reflect reality, their model does incorporate real historical data such as Cro-Magnon expansion over time and local population growth. At a value of only 0.1%, their new estimate of the rate of interbreeding is about 400 times lower than previous estimates and provides strong support that Neanderthals and Cro-Magnon didn't interbreed and may even have been different species.

