# Correction: Cro-Magnons Conquered Europe, but Left Neanderthals Alone

**DOI:** 10.1371/journal.pbio.0030090

**Published:** 2005-02-15

Published February 15, 2005

In *PLoS Biology*, volume 2, issue 12.

10.1371/journal.pbio.0020449

The art credits were missing from the image accompanying this synopsis. The image caption should read as follows:[Fig pbio-0020449-g001]

**Figure pbio-0020449-g001:**
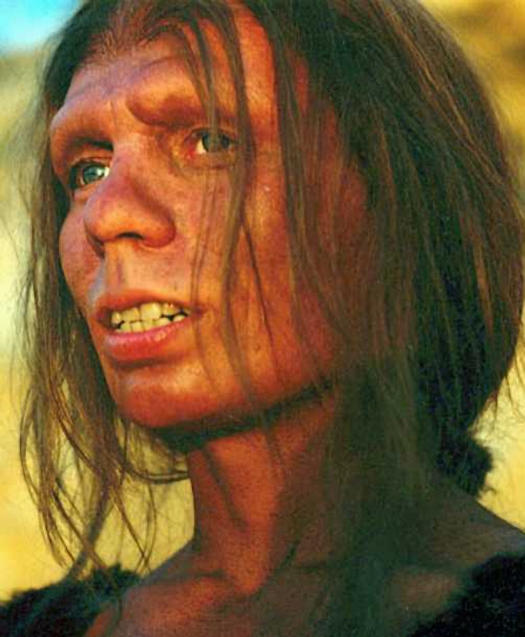
Reconstruction of Neanderthal woman (Photo: Bacon Cph; makeup: Morton Jacobsen)

